# High-resolution melting analysis for molecular detection of multidrug resistance tuberculosis in Peruvian isolates

**DOI:** 10.1186/s12879-016-1615-y

**Published:** 2016-06-09

**Authors:** Marco Galarza, Manuel Fasabi, Kelly S. Levano, Edith Castillo, Nadia Barreda, Mitzi Rodriguez, Heinner Guio

**Affiliations:** Laboratorio de Biotecnología y Biología Molecular, Instituto Nacional de Salud, Lima, Peru; Laboratorio de Referencia Regional de Tuberculosis y Microbiología, DIRESA Callao, Lima, Peru

**Keywords:** Tuberculosis, High Resolution Melting Analysis, Multi-drug resistance tuberculosis

## Abstract

**Background:**

The emergence of multidrug-resistant strains is a major health problem especially for countries with high TB incidence such as Peru. In this study, we evaluated High Resolution Melting (HRM) assay in Peruvian isolates for the detection of mutations within *rpoB*, *katG* genes and promoter region *inhA* to determine isoniazid and rifampicin resistance in *Mycobacterium tuberculosis* (*Mtb*).

**Methods:**

DNA samples extracted from a total of 167 clinical isolates of *Mtb*, 89 drug-sensitive and 78 multidrug-resistant, were blindly analyzed by HRM analysis and verified by DNA sequencing.

**Results:**

The HRM analysis generated patterns that were specific to distinguish between sensitive and resistance isolates. The sensitivity and specificity of the HRM assays in comparison with drug susceptibility testing (DST) for detection of rifampicin resistance were 98.7 % and 97.5 %, and for isoniazid resistance were 98.7 % and 100 %.

**Conclusion:**

This study suggests that HRM Analysis could help with rapid diagnosis of MDR-TB cases in Peru.

## Background

In countries with high TB burden such as Peru, the emergence of multidrug-resistant strains is a major health problem. In 2013, Peru registered 27 504 new TB cases (21 916 pulmonary and 5588 extrapulmonary), from which a 3.9 % (850) were multidrug-resistant tuberculosis (MDR-TB) [1]. MDR-TB is defined as resistance to rifampicin and isoniazid, two of the first-line anti-TB drugs [1]. According to the WHO TB report, research should focus on the development of simple and rapid diagnostic methods that can be used at first level health facilities to diagnose and thus prevent the increase of MDR-TB cases [2]. The conventional phenotypic drug susceptibility testing (DST) method, Agar Proportion Method (APM) continues to be the gold standard for *Mtb* DST. The problem with the current conventional DST methods available is that they are generally carried out after a culture is isolated from a clinical sample detecting *Mtb*’s growth in the presence of anti-TB drugs by absolute concentration, resistance ratio or portion methods on solid medium having a turnaround time of about 2–4 weeks. Therefore, more rapid TB DST methods that can use clinical samples directly are needed to give a prompt diagnosis. Some non-commercially available direct methods such as MODS (microscopic observation drug susceptibility) assay have been developed. However, the MODS assay, which can process a large number of sputum samples with results in 7–9 days, is too laborious and time consuming due to the need of daily plate readings [3]. There has been an increased effort to develop new simple and rapid diagnostic molecular methods for MDR-TB taking advantage of mutations that render resistance [4]. For rifampicin, resistance is observed with the presence of localized mutations in the 81 bp region of the *rpoB* gene [5]. Also, mutations in the *rpoA* and *rpoC* regions have been recently reported in resistant strains and would be important in future diagnostic methods [6, 7]. Resistance to isoniazid is observed mostly with the presence of mutations in *katG*. Other important mutations also occur in *ahpC*, *kasA*, *ndh* genes and the promoter region of *inhA* [8–11]. The current molecular methods can detect resistance-associated mutations within 1–2 days using clinical samples. One of the commercially available molecular methods is Xpert MTB/RIF (Cepheid), a real-time PCR assay that detects rifampicin resistance in sputum samples. There are also two commercially available DNA macroarray assays (reverse hibridization-based line probe assays), INNO-LiPA rif (Innogenetics), which detect mutations on *rpoB* gene, and MTBDR*plus* (Hain Lifesciences), which simultaneously detects rifampicin (*rpoB* gene) and isonizid (*katG* and *inhA* region) resistance [12, 13]. These assays are able to reduce the time for MDR-TB diagnosis. In our quest to develop another molecular method that can be helpful for diagnosing MDR-TB and be implemented in Peru, we evaluated the HRM assay. Some of the advantages of HRM analysis include short processing time, simple procedure and low cost compared to current methods used in Peru [14]. HRM is based on the analysis of fluorescence curves produced by DNA intercalating dye during strand dissociation events in the melting phase following real-time PCR. It allows the detection of genetic mutations or variance in nucleic acid sequences [15]. The use of this methodology include identification of SNPs, genotyping, gene scanning, sequence matching and nucleic acids methylation [16]. In this study, we evaluated the detection of mutations in *rpoB*, *katG* genes and the *inhA* promoter region by HRM analysis using Peruvian culture isolates with known phenotypic susceptibility profiles of MDR-TB.

## Methods

### Sample collection

DNA samples extracted from a total of 167 *Mtb* isolates were used for this study. Convenience sampling was applied. The isolates were provided by the Laboratory Reference of Mycobacteria from DIRESA Callao, province with one of the highest number of TB cases in PERU. 89 isolates had sensitive phenotype (susceptible to both rifampicin and isoniazid) and 78 isolates had multidrug-resistant phenotype (resistant to both rifampicin and isoniazid), as determined by MODS. MODS assay was performed as described previously [17, 18] using critical concentrations of 0.4 μg/mL for isoniazid and 1.0 μg/mL for rifampicin. All but 14 isolates that had a resistant phenotype were verified by APM in the Reference Laboratory of Mycobacteria at the Instituto Nacional de Salud. Briefly, APM was performed in accordance with WHO guidelines [19] on Middlebrooks 7H10 agar containing 0.2 μg.ml and 1 μg/ml for INH or 1 μg/ml for RIF. The MODS and APM results were concordant in all samples analyzed. 64 % of the resistant samples had high level INH resistance and 11.5 % had low-level INH resistance. 17.9 % of the resistant samples had no APM results available (some due to their inability to grow on agar. For those, a molecular testing method was done). All the isolates that had the high level INH resistance and only one had both katG and inhA mutations, had katG mutations, and all the isolates that had low level INH resistance had inhA mutations.

All other processes and experimental methodologies were performed in the Biotechnology and Molecular Biology Laboratory at the Instituto Nacional de Salud (INS), Chorrillos. This project was approved by the INS‘s Research and Ethical Committees (OI-017-13) and has the participants’ consent to publish these data.

### Extraction of DNA

Bacteria, grown on Lowenstein Jensen solid medium, were resuspended in molecular biology grade water and sonicated using S2 ultrasonicator (Covaris Inc, USA). The bacterial solution was then treated with lysozyme overnight. Finally, the DNA was extracted using the PureLink™ Genomic DNA kit (ThermoFisher Scientific, USA) according to manufacturer’s procedure.

### High resolution melting analysis

HRM assays were performed using Type I HRM PCR kit (Qiagen). All samples were tested in duplicates. The primers used for these experiments were previously reported [20] (Table 1). For confirmation of *Mtb*, the IS6110 primers were used. Each reaction included: 1X HRM master mix (containing HotStarTaq *Plus* DNA polymerase, HRM PCR buffer with EvaGreen dye, Q-solution and dNTPs), 0.7uM of each primer, 2 ul (1 ng/ul) of *Mtb* DNA, and RNase-Free water. All assays were run on the Rotor-Gene Q real time PCR instrument (Qiagen, USA) using the following cycling parameters: initial denaturation at 95 °C for 10 min, 45 cycles of 94 °C for 30 s and 60 °C for 40 s. For HRM analysis, a second hold was set at 60 °C for 1 min to allow reannealing of the amplified DNA. The melt analysis was performed from 80–94 °C with 0.1 °C increments every 2 s. The reference strain H37Rv, susceptible to RIF and INH, was included in each run as a wild-type positive control and nuclease free water was used as a negative control. The biologists who performed the HRM analysis were blinded to the phenotype and genotype resistance data.

**Table 1:** Sequences of the primers used HRM analysis

Primer	Target	Sequence	Fragment size (bp)	HRM temperature range (°C)
IS6110-F		TCCGGCCGGTGGTCG		
	*IS6110*		103	
IS6110-R		CGGCGCTTGTGGGTCAA		
rpoB-F		CGCGATCAAGGAGTTCTTC		
	*rpoB*		118	
rpoB-R		TGACAGACCGCCGGGCCC		80–95
katG-F		GCTGAGCCAATTCATGGACC		
	*katG*		235	
katG-R		TGACAGACCGCCGGGCCC		
inhA-F		GCGGTCACACTTTCGGTAA		
	Promoter *inhA*		180	
inhA-R		GGTGTTCGTCCATACGACCT		

*F* forward primer; *R* reverse primer

The HRM data were analyzed with the Rotor-Gene Q software. The melting curves generated represented by different color lines and shapes easily differentiate between sensitive and resistant isolates.

### DNA sequencing

Sequencing analysis was used to confirm resistance in all phenotypically resistant strains using the primers listed on Table 2, which have been previously reported (16). Sequencing was performed using 3500 Genetic Analyzer (Applied Biosystems, USA), and subsequent alignment was done using Geneious 5.4.4 program.

**Table 2:** Sequence of primers used DNA sequencing

Primer	Gene	Sequence	Fragment size (bp)
rpoB-FS		TGGTCCGCTTGCACGAGGGTCAGA	
	*rpoB*		758
rpoB-RS		CAGGAAGGGAATCATCGCGG	
katG-FS		TGCAGATGGGGCTGATCTACG	
	*katG*	ACCCATGTCTCGGTGGATCAG	596
katG-RS			
inhA-FS		ACATACCTGCTGCGCAAT	
	*inhA*	TCACATTCGACGCCAAAC	400
inhA-RS			

*F* forward; *R* reverse

### Statistical analysis

The sensitivity and specificity parameters were calculated before and after discrepancies were resolved using statistical formula. Sensitivity was calculated as follows: (true positive/(true positive + false negative); specificity was calculated as follows: (true negative)/(true negative + false positive). The data were analyzed using the EPIDAT ver. 3.1 software. The study conforms to the international standards for reporting of diagnostic accuracy (STARD) requirements [21].

## Results and Discussion

### Detection of rifampicin resistance using HRM analysis

**Fig. 1:**
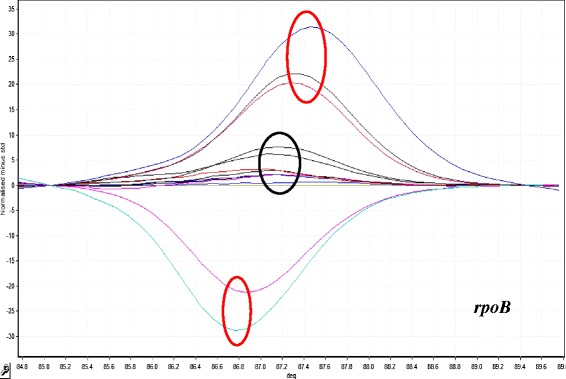
HRM curve analysis for resistant discrimination in 118 bp amplified fragment from rpoB gene. Resistant profiles with positive and negative fluorescence are shows in red circles (five samples). Sensitive profiles are shows in black circle (seven samples)

To evaluate the ability of the HRM assay to detect rifampicin resistance, we analyzed sequence variations in a 118 bp region of the *rpoB* gene on the DNA samples of 167 *Mtb* isolates. 89 isolates were sensitive and 78 were MDR as determined by MODS and confirmed by APM. According to the HRM results, there were phenotypic and genotypic (HRM analysis) concordance for 77 out of 78 resistant isolates. One phenotypic resistant isolate was genotypic sensitive and two phenotypic sensitive isolates were genotypic resistant by HRM (Fig. 1).

### Detection of isoniazid resistance using HRM analysis

**Fig. 2:**
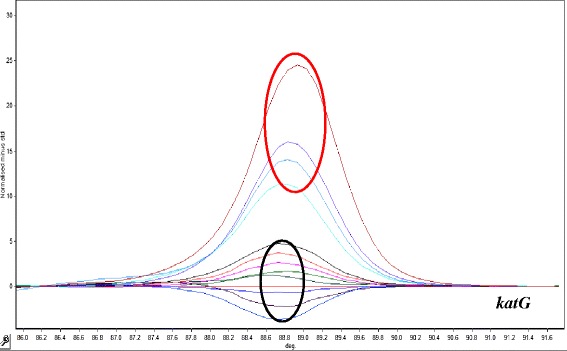
HRM curve analysis for resistant discrimination in 235 pb amplified fragment from katG gene. Resistant profiles are show in red circle (four samples). Sensitive profiles are show in black circle (eight samples)

**Fig. 3:**
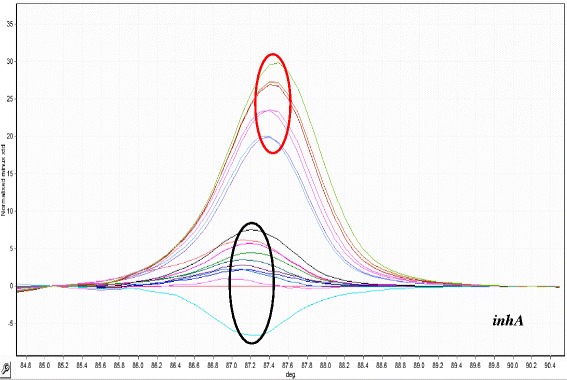
HRM curve analysis for resistant discrimination in 190 pb amplified fragment from inhA promotor region. Resistant profiles are show in red circle (seven samples). Sensitive profiles are show in black circle (ten samples)

To evaluate the ability of the HRM assay to detect isoniazid resistance, we analyzed sequence variations in a 235 bp region of the *katG* gene and in a 190 bp region of the *inhA* promoter the DNA samples of the 167 isolates. The results of the HRM analysis showed that there was phenotypic and genotypic (HRM analysis) concordance for 77 out of 78 resistant isolates. One phenotypic resistant isolate was genotypic sensitive. HRM analysis for *katG* gene detected 90 sensitive and 67 isoniazid resistant isolates (Fig. 2), and for the promoter region of *inhA* 90 sensitive and 10 isoniazid resistant isolates were detected (Fig. 3).

### DNA sequencing

Seventy-eight samples with drug-resistant profiles were sequenced for the *rpoB*, *katG* and *inhA* targets (Table 3). Fourteen types of mutations were observed in the *rpoB* gene of the resistant samples, with S531L (TCG➔TTG) being the most common. In the *katG* gene, the most common mutation was S315T, and C → T (−15) for *inhA* promoter. All the mutations observed have been previously linked to resistance to INH and RIF.

**Table 3:** Results of HRM curves analysis for *rpoB*, *katG* and *inhA* targets in *Mycobacterium tuberculosis*

DRUG	PHENOTYPE	TARGET	N°	MUTATION	HRM CURVE ASSAY	DNA SEQUENCING
RIFAMPICIN	Resistant (78)	*rpoB*	53	S531L (TCG → TTG)	mutant	mutant
			14	D516V (GAC → GTC)	mutant	mutant
			2	H526D (CAC → GAC)	mutant	mutant
			1	Q513L (CAA → CTA)	mutant	mutant
			1	Q513P (CAA → CCA)	mutant	mutant
			1	M515I (ATG → ATA), D516G (GAC → GGC)	mutant	mutant
			1	D516Y (GAC → TAC), N518H (AAC → CAC)	mutant	mutant
			1	H526R (CAC → CGC)	mutant	mutant
			1	H526Y (CAC → TAC)	mutant	mutant
			1	S531F (TCG → TTT)	mutant	mutant
			1	S531W (TCG → TGG)	mutant	mutant
			1	No mutation	wild	wild
	Susceptible (89)		87	Not sequenced	wild	-
			2	514 Phe (TTC → TTT)	mutant	wild
ISONIAZID	Resistant (78)	*katG*	66	S315T (AGC → ACC)	mutant	mutant
			1	S315T (AGC → ACC), C → T (−15)	mutant	mutant
		*inhA*	10	C → T (−15)	mutant	mutant
		*katG,inhA*	1	No mutation	wild	wild
	Susceptible (89)		87	Not sequenced	wild	-
			2	No mutation	wild	wild

Two samples with a sensitive phenotype that turn out resistant by HRM analysis were also sequenced. Sequencing revealed a synonymous mutation in codon 514 (TTC➔TTT), reason why the sample has a sensitive phenotype. A drug-resistant sample that gave wild-type results with HRM analysis had no mutations in *rpoB*, *katG* and *inhA* promoter, suggesting the possibility of mutations in other genes (like *rpoA*, *rpoC*, *ahpC*, *kasA* and *ndh*).

### Analysis of sensitivity and specificity of HRM analysis

Most of HRM analysis results were consistent with the resistant or sensitive phenotype. The sensitivity for detection of rifampicin resistance was found to be 98.7 % and the specificity 97.75 %. For isoniazid resistance (*katG* and/or *inhA*), the sensitivity was found to be 98.7 % and the specificity 100 % (Table 4).

**Table 4:** Sensitivity and specificity of HRM analysis in comparison with Drug susceptibility

Drug	Target	HRM profile		% variant	Drug susceptibility		% resistant	Sensitivity (%)	Specificity (%)
		+	−		+	−			
Rifampicin	*rpoB*	79	88	47.3	78	89	46.7	98.7 (92.2–99.9)	97.75 (91.2–99.6)
Isoniazid	*katG* and/or	77	90	46.1	78	89	46.7	98.7 (92.2–99.9)	100 (94.2–99.9)
	*inhA* promoter								

## Conclusions

At present, a rapid and effective diagnosis for TB and MDR-TB is necessary to provide a better treatment for infected individuals. Molecular biology techniques like HRM analysis can be employed to detect MDR-TB due to their high sensitivity, specificity and efficiency [20, 22, 23]. In this study, we evaluated the performance of HRM curve analysis as a method for the rapid and accurate detection of mutations associated to MDR-TB. All isolates with drug-resistance profiles were confirmed by DNA sequencing analysis. In comparison with previous studies, our results of sensitivity and specificity are similar to others (24–26). One limitation of the current study is that the target region used for the HRM analysis does not cover all mutations related to resistance, and thus, the technique cannot yet replace conventional DST. Another limitation is that the test may detect silent or other mutations not actually associated with drug resistance. An advantage of this technique is that it can be implemented to detect fluoroquinolone, pyrazinamide and streptomycin resistance [24–26].

In conclusion, this study showed that using the rpoB, katG genes and inhA promoter region as targets in the HRM assay can be used to detect MDR-TB in culture isolates with high sensibility and specificity. Evaluation of the HRM assay on sputum samples would be an important next step to further expedite MDR-TB detection.

## Abbreviations

HRM, high resolution melting; TB, tuberculosis; *Mtb*, *Mycobacterium tuberculosis*; DST, drug susceptibility testing; APM, Agar Proportion Method; MDR-TB, multidrug-resistance tuberculosis; MODS, Microscopic observations drug susceptibility

## References

[1] WHO: World Health Organization's global TB database: Peru 2014. In*.*

[2] WHO: The global plan to stop TB 2011–2015: transforming the fight towards elimination of tuberculosis. 2010.

[3] Siddiqi S, Ahmed A, Asif S, Behera D, Javaid M, Jani J, et al. Direct drug susceptibility testing of Mycobacterium tuberculosis for rapid detection of multidrug resistance using the Bactec MGIT 960 system: a multicenter study. J Clin Microbiol. 2012;50(2):435–40. 10.1128/JCM.05188-11 PMC3264138 22162558

[4] Moscoso MY, Jave HO, Rojas MC, Gutiérrez C, Romaní FR (2013). Agenda Nacional de Investigación en Tuberculosis en Perú, 2011–2014. Rev Panam Salud Publica.

[5] Brandis G, Wrande M, Liljas L, Hughes D (2012). Fitness‐compensatory mutations in rifampicin‐resistant RNA polymerase. Mol Microbiol.

[6] De Vos M, Müller B, Borrell S, Black P, Van Helden P, Warren R, Gagneux S, Victor T (2013). Putative compensatory mutations in the rpoC gene of rifampin-resistant Mycobacterium tuberculosis are associated with ongoing transmission. Antimicrob Agents Chemother.

[7] Comas I, Borrell S, Roetzer A, Rose G, Malla B, Kato-Maeda M, Galagan J, Niemann S, Gagneux S (2012). Whole-genome sequencing of rifampicin-resistant Mycobacterium tuberculosis strains identifies compensatory mutations in RNA polymerase genes. Nat Genet.

[8] Ando H, Kondo Y, Suetake T, Toyota E, Kato S, Mori T, Kirikae T (2010). Identification of katG mutations associated with high-level isoniazid resistance in Mycobacterium tuberculosis. Antimicrob Agents Chemother.

[9] Cardoso RF, Cardoso MA, Leite CQF, Sato DN, Mamizuka EM, Hirata RDC, Mello FFd, Hirata MH. Characterization of ndh gene of isoniazid resistant and susceptible Mycobacterium tuberculosis isolates from Brazil. Mem Inst Oswaldo Cruz. 2007;102(1):59–61. 10.1590/s0074-02762007000100009 17294000

[10] Rindi L, Bianchi L, Tortoli E, Lari N, Bonanni D, Garzelli C (2005). Mutations responsible for Mycobacterium tuberculosis isoniazid resistance in Italy. Int J Tuberc Lung Dis.

[11] Somoskovi A, Parsons LM, Salfinger M (2001). The molecular basis of resistance to isoniazid, rifampin, and pyrazinamide in Mycobacterium tuberculosis. Respir Res.

[12] Ahmad S, Mokaddas E (2014). Current status and future trends in the diagnosis and treatment of drug-susceptible and multidrug-resistant tuberculosis. J Infect Public Health.

[13] Ahmad S, Mokaddas E (2009). Recent advances in the diagnosis and treatment of multidrug-resistant tuberculosis. Respir Med.

[14] Solari L, Gutiérrez A, Suárez C, Jave O, Castillo E, Yale G, Ascencios L, Quispe N, Valencia E, Suárez V (2011). Análisis de costos de los métodos rápidos para diagnóstico de tuberculosis multidrogorresistente en diferentes grupos epidemiológicos del Perú. Rev Peru Med Exp Salud Publica.

[15] Lee AS, Ong DC, Wong JC, Siu GK, Yam W-C (2012). High-resolution melting analysis for the rapid detection of fluoroquinolone and streptomycin resistance in Mycobacterium tuberculosis. PLoS One.

[16] Montgomery JL, Sanford LN, Wittwer CT (2010). High-resolution DNA melting analysis in clinical research and diagnostics. Expert Rev Mol Diagn.

[17] Caviedes L, Lee T-S, Gilman RH, Sheen P, Spellman E, Lee EH, Berg DE, Montenegro-James S (2000). Rapid, efficient detection and drug susceptibility testing of Mycobacterium tuberculosis in sputum by microscopic observation of broth cultures. J Clin Microbiol.

[18] Park WG, Bishai WR, Chaisson RE, Dorman SE (2002). Performance of the microscopic observation drug susceptibility assay in drug susceptibility testing for Mycobacterium tuberculosis. J Clin Microbiol.

[19] World Health Organization (2008). Drug susceptibility testing, proportion method.

[20] Chen X, Kong F, Wang Q, Li C, Zhang J, Gilbert GL (2011). Rapid detection of isoniazid, rifampin, and ofloxacin resistance in Mycobacterium tuberculosis clinical isolates using high-resolution melting analysis. J Clin Microbiol.

[21] Bossuyt PM, Reitsma JB, Bruns DE, Gatsonis CA, Glasziou PP, Irwig LM, Moher D, Rennie D, de Vet HC, Lijmer JG (2003). The STARD statement for reporting studies of diagnostic accuracy: explanation and elaboration. The Standards for Reporting of Diagnostic Accuracy Group. Croat Med J.

[22] Ramirez MV, Cowart KC, Campbell PJ, Morlock GP, Sikes D, Winchell JM, Posey JE (2010). Rapid detection of multidrug-resistant Mycobacterium tuberculosis by use of real-time PCR and high-resolution melt analysis. J Clin Microbiol.

[23] Yadav R, Sethi S, Mewara A, Dhatwalia S, Gupta D, Sharma M (2012). Rapid detection of rifampicin, isoniazid and streptomycin resistance in Mycobacterium tuberculosis clinical isolates by high‐resolution melting curve analysis. J Appl Microbiol.

[24] Pholwat S, Stroup S, Gratz J, Trangan V, Foongladda S, Kumburu H, Juma SP, Kibiki G, Houpt E (2014). Pyrazinamide susceptibility testing of Mycobacterium tuberculosis by high resolution melt analysis. Tuberculosis.

[25] Pang Y, Zhou Y, Wang S, Lu J, Lu B, He G, Wang L, Zhao Y (2011). A novel method based on high resolution melting (HRM) analysis for MIRU–VNTR genotyping of Mycobacterium tuberculosis. J Microbiol Methods.

[26] Issa R, Abdul H, Hashim SH, Seradja VH, Shaili NA, Hassan NAM (2014). High resolution melting analysis for the differentiation of Mycobacterium species. J Med Microbiol.

